# Catalytic Reductive Amination of Aldehydes and Ketones With Nitro Compounds: New Light on an Old Reaction

**DOI:** 10.3389/fchem.2020.00215

**Published:** 2020-04-15

**Authors:** Alexey Yu. Sukhorukov

**Affiliations:** ^1^Laboratory of Organic and Metal-organic Nitrogen-Oxygen Systems, N. D. Zelinsky Institute of Organic Chemistry, Moscow, Russia; ^2^Department of Innovational Materials and Technologies Chemistry, Plekhanov Russian University of Economics, Moscow, Russia

**Keywords:** nitro compounds, reductive amination, secondary amines, heterogeneous catalysis, cyclizations, nitrogen heterocycles, pharmaceuticals

## Abstract

Reductive amination of carbonyl compounds with primary amines is a well-established synthetic methodology for the selective production of unsymmetrically substituted secondary and tertiary amines. From the industrial and green chemistry perspective, it is attractive to combine reductive amination with the synthesis of primary amines in a single one-pot catalytic process. In this regard, nitro compounds, which are readily available and inexpensive feedstocks, received much attention as convenient precursors to primary amines in such processes. Although the direct reductive coupling of nitro compounds with aldehydes/ketones to give secondary and tertiary amines has been known since the 1940's, due to the development of highly efficient and selective non-noble metal-based catalysts a breakthrough in this area was made in the last decade. In this short overview, recent progress in the methodology of the reductive amination with nitro compounds is summarized together with applications to the synthesis of bioactive amines and heterocycles. Remaining challenges in this field are also analyzed.

## Introduction

Secondary amines are privileged compounds in the design of pharmaceutically relevant molecules, as well as important building blocks in the synthesis of agrochemicals, dyes, and functional materials. However, direct synthesis of secondary amines by double alkylation of ammonia is problematic because of its low selectivity and environmental issues. Preparation of unsymmetrically substituted secondary amines by selective monoalkylation of primary amines is even more challenging. The selectivity issue is efficiently solved by the reductive amination process, in which controlled alkylation of primary amines is performed by condensation with aldehyde/ketone to form an imine followed by the reduction of the C=N bond (usually by catalytic hydrogenation or hydride transfer) (Tarasevich and Kozlov, 1999; Afanasyev et al., 2019). From the viewpoint of green chemistry, it is beneficial to combine reductive amination with the synthesis of primary amines is a one-pot process. This strategy is efficiently realized by using nitroarenes and nitroalkanes as convenient sources of aromatic and aliphatic amines (Orlandi et al., 2018).

Nitro compounds are readily available feedstocks (Green and Johnson, 2000). Simple aliphatic nitro compounds are prepared by Konovalov nitration of alkanes on an industrial scale as well as by the radical nitration of C–H active compounds (Ono, 2001). Kormblum reaction (nucleophilic substitution of halide for the nitrite ion) and the addition of electrophiles to nitronate anions are widely used to prepare branched and functionalized nitro compounds of aliphatic series (Ono, 2001). Nitroarenes are easily accessed through the electrophilic nitration of C–H, C–B, C–M, and C–Hal bonds in arenes (Olah and Malhotra, 2001; Yan and Yang, 2013). Furthermore, both aliphatic and aromatic nitro compounds can be delivered from the oxidation of various nitrogen-containing derivatives, such as nitroso compounds, oximes, azides, etc. (Ono, 2001).

Conventional reductive amination protocols utilizing mild hydride-based reagents (Abdel-Magid et al., 1996) are not suitable for the reduction of the nitro group. For this reason, catalytic hydrogenation is used to transform NO_2_ fragment into the amino group and perform subsequent reductive amination under the same conditions in a tandem sequence. In this short overview, recent advances in the methodology and application of the catalytic reductive amination with nitro compounds are outlined.

## Intermolecular Reductive Amination of Aldehydes and Ketones With Nitro Compounds: Recent Progress

**Figure 1:**
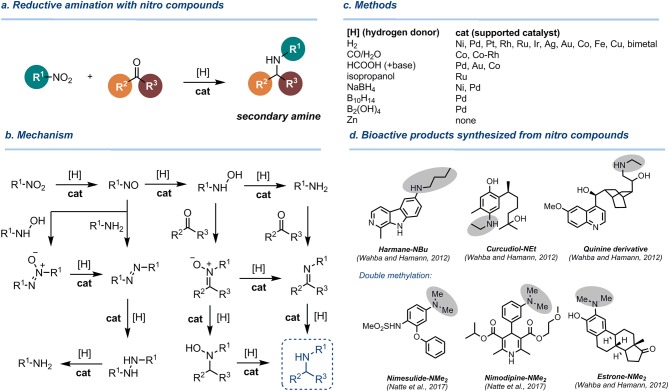
Reductive amination of aldehydes and ketones with nitro compounds. **(a)** Overall reaction scheme. **(b)** Mechanism. **(c)** Methods. **(d)** Bioactive products synthesized by reductive alkylation of nitro compounds.

Studies on the reductive amination of carbonyl derivatives with nitro compounds (reductive alkylation of nitro compounds) date back to the first half of twentieth century (Major, 1931) (Figure 1a). In 1940, Emerson and Mohrman reported on the coupling of aromatic nitro compounds with aldehydes under catalytic hydrogenation over Raney nickel catalyst (cat = Ra-Ni) (Emerson and Mohrman, 1940). Although the procedure was general for both aromatic and aliphatic aldehydes, the yields of secondary amines were moderate in many cases.

The reasons for low selectivity in this and other catalytic protocols are underlay by the complexity of the mechanism of the nitro group reduction involving numerous reactive intermediates (nitroso derivatives, hydroxylamines, azocompounds, imines, oximes, etc., Figure 1b), which can be isolated as side-products (Benchekroun-Mounir et al., 1993; Maeda et al., 1999). These intermediates can react with carbonyl compounds or primary amines, leading to side processes and a decrease of yield of target secondary amines. Other selectivity issues arise from the hydrogenation of the aromatic ring (Cirujano et al., 2013) and the formation of tertiary amines by a double reductive amination (Emerson and Uraneck, 1941). In reactions with formaldehyde, double reductive methylation to give tertiary amines occurs very easily, while the controlled monomethylation is challenging (Natte et al., 2017).

The nature of catalyst, support, and additives is highly important to ensure full conversion of the aforementioned intermediates and for achieving selective synthesis of secondary amines from nitro compounds. The application of supported platinum catalysts was shown to be advantageous in terms of selectivity and yield of target secondary amines. The platinum-catalyzed process for reductive alkylation of nitroarenes developed by Bayer could be used for a semi-industrial production of *N*-alkyl-*N*-aryl amines (Maurer et al., 1998). Modification of platinum catalysts by various acidic additives increases the selectivity and yield of secondary amines by preventing degradation of amines through the formation of salts (Tarasevich and Kozlov, 1999).

In the last decade, numerous other catalytic systems were developed based on supported palladium (Sydnes et al., 2008; Sreedhar et al., 2009; Dell'anna et al., 2011; Wei et al., 2013; Zhou et al., 2015; Sharma et al., 2017), platinum (Hu et al., 2011; Cirujano et al., 2013), rhodium (Huang et al., 2015), iridium Pintado-Sierra et al., 2013; Sui et al., 2017), ruthenium (Del Pozo et al., 2012), silver (Artiukha et al., 2017), and gold (Yamane et al., 2009; Artiukha et al., 2015) nanoparticles. Heterobimetallic systems such as Pd-Ag (Li et al., 2013; Chen et al., 2015), Pd-Au (Cho et al., 2018; Yin et al., 2018), Ni-Pd (Nişanci et al., 2015), and Rh-Co (Park and Chung, 2015) were also shown to be efficient catalysts for the reductive alkylation of nitro compounds. With noble metal catalysts, reductive alkylation of nitro compounds is performed under mild conditions (r.t. to 100°C) and low hydrogen pressure (typically, 1–25 bar). Palladium and platinum-catalyzed processes are characterized by high TON (up to 3,800, average ca. 100÷200) and TOF (up to 1,900, average ca. 50÷100 h^−1^) values (Cirujano et al., 2013; Kalbasi and Mazaheri, 2016). Furthermore, these catalysts often show very good recyclability (Park and Chung, 2015; Cho et al., 2018). However, noble metal catalysts are not always selective for the reductive *N*-alkylation of nitroarenes with aromatic aldehydes. Benzyl alcohols and methylarenes resulting from the hydrogenolysis of aromatic aldehydes are usually found as the by-products with platinum metal-based catalysts (Huang et al., 2015). Another limitation is the inability to tolerate functional groups reducible under hydrogenation over platinum metal catalysts (C–I, C–Br, C–S, double and triple bonds, azido group, etc.). Few examples of palladium-catalyzed methods tolerating bromoarenes and cyano-group were reported (Bae et al., 2000; Kalbasi and Mazaheri, 2016; Sharma et al., 2017). With silver and gold catalysts, bromoarene and alkene fragments are retained in the resulting amine product (Zhang et al., 2016; Artiukha et al., 2017).

Considerable efforts have been made toward finding non-noble metal catalysts to replace noble metals in the reductive amination of carbonyl compounds with nitro compounds. Supported catalysts based on nickel (Fiore et al., 2019; Li et al., 2019), cobalt (Stemmler et al., 2014; Jagadeesh et al., 2017; Senthamarai et al., 2018), copper (Nuzhdin et al., 2017), and iron (Natte et al., 2017) nanoparticles were developed. An important advantage of non-noble metal catalyzed reactions is their tolerance to C–I and C–S bonds, which undergo hydrogenolysis with palladium/platinum catalysts (Stemmler et al., 2014; Natte et al., 2017; Pedrajas et al., 2017; Fiore et al., 2019). However, harsh conditions (110–170°C and 50–70 bar H_2_), long reaction times (10–40 h), and high catalyst loadings (average TON 25÷50) are usually required with non-noble metal catalysts. Recently, Beller and Llusar reported that a well-defined molybdenum sulfide cluster Mo_3_S_4_ exhibits exceptionally high catalytic activity allowing for the performance of reductive benzylation and alkylation of aromatic and aliphatic nitro compounds at 70°C and 20–50 bar H_2_ with >99% conversion and high yields (Pedrajas et al., 2017). Unfortunately, this catalyst showed poor recyclability.

Although most of the catalytic protocols for reductive amination with nitroarenes were designed for batch reactors, flow methods using supported gold and silver catalysts have been reported in recent years (Artiukha et al., 2015, 2017).

Numerous studies were performed to replace hydrogen for other reducing agents, in particular those which are easy-to-use in the lab (Figure 1c). As an example, Rhee developed reductive amination of aldehydes with nitroarenes using ammonium formate over Pd/C catalyst (Byun et al., 2007). However, this method was not applicable to aromatic aldehydes. Cao and co-workers developed reductive amination of aldehydes with nitroarenes using formic acid as a transfer hydrogenation reagent and gold catalyst supported on rutile titania (Au/TiO_2_-R) under very mild conditions (80°C, 1 bar) in water (Zhang et al., 2016). Li and co-workers accomplished reductive benzylation of nitrobenzene with formic acid over Pd on graphitic carbon nitride (g-C_3_N_4_) catalyst at 100°C, yet the substrate scope was not studied (Xu et al., 2018). Zhou and Zhang successfully performed reductive alkylation of nitro compounds with HCOOH over Co-N_x_ catalyst (Zhou and Zhang, 2017) or Co nanoparticles embedded in mesoporous nitrogen-doped carbon (Jiang et al., 2017; Zhou et al., 2017b). Very recently, Chen and co-workers used N,S-codoped carbon shell-enclosed ultrafine Co nanoparticles to catalyze the same reaction (Guo et al., 2019). These Co-catalyzed transfer hydrogenation protocols are characterized by a broad functional group tolerance, yet harsh conditions were required (up to 190°C) to achieve full conversion. As a result, formylation of the amino group takes place as a side process in transfer hydrogenation processes with HCOOH at elevated temperatures (Guo et al., 2019).

Jagadeesh developed an efficient catalytic system based on a nitrogen-doped, graphene-activated nanoscale Co_3_O_4_-based catalyst with HCOOH-Et_3_N as a hydrogen source (Senthamarai et al., 2018). Importantly, not only aromatic and aliphatic aldehydes, but also formaldehyde could be involved in the reductive coupling with nitro compounds under these conditions. Transfer hydrogenation with a CO/H_2_O system with supported Co-nanoparticles (Zhou et al., 2017a) and cobalt-rhodium heterobimetallic nanoparticle catalysts (Park and Chung, 2015) was also used with a similar efficacy and substrate scope. Several reports deal with the use of NaBH_4_ as a mild, inexpensive, and safe reducing agent in the presence of nickel (Kalbasi and Mazaheri, 2015; Fiore et al., 2019) or palladium nanoparticles (Kalbasi and Mazaheri, 2016). Decaborane (B_10_H_14_) (Bae et al., 2000) and diboronic acid (B_2_(OH)_4_) (Zhou Y. et al., 2017) in combination with Pd/C catalyst were also shown to be convenient systems for reductive alkylation of nitroarenes with aromatic and aliphatic aldehydes. Non-catalytic methods utilizing zinc as a reducing agent have also been reported (Zou et al., 2007; Jiang et al., 2009; Wahba and Hamann, 2012; Lin et al., 2016), yet their discussion is beyond the scope of this short review.

Reductive alkylation of nitro compounds was shown to be a useful methodology for the synthesis of pharmacologically active ingredients and bioactive molecules (Figure 1d). Hamann et al. successfully used this strategy to synthesize modified natural molecules (alkaloids, sesquiterpenes, and steroids), possessing secondary and tertiary amino groups (Wahba and Hamann, 2012). In a similar fashion, Beller and co-workers utilized iron-catalyzed reductive methylation of nitro compounds to perform the post-modification of pharmaceuticals and fluorescent molecules (Natte et al., 2017).

## Reductive Alkylation of Nitro Compounds in the Synthesis of Heterocycles: Characteristic Examples

**Figure 2:**
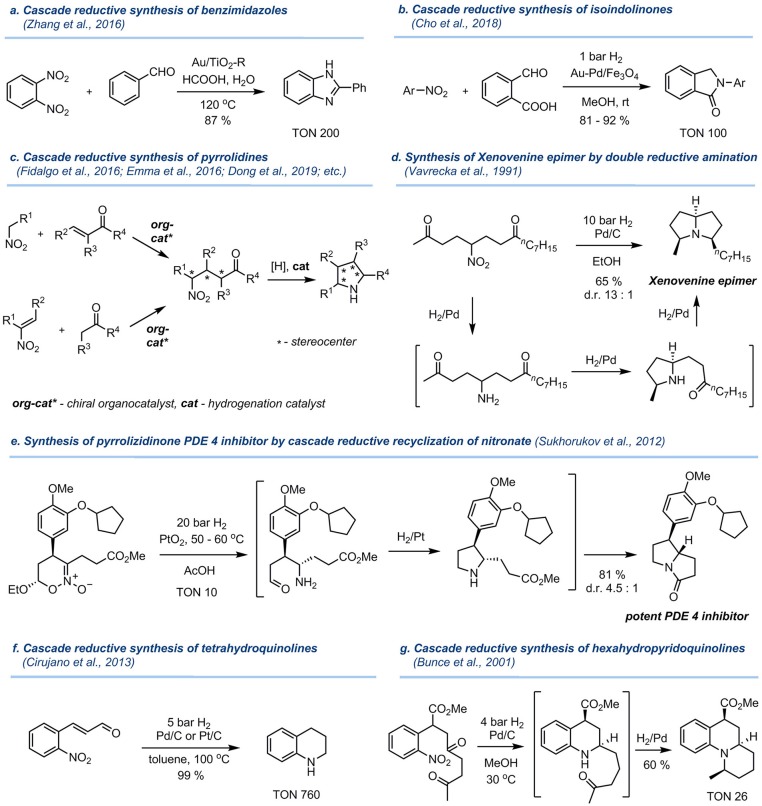
Examples of a cascade synthesis of bioactive heterocycles by reductive amination/cyclization involving nitro compounds. **(a)** Cascade reductive synthesis of benzimidazoles. **(b)** Cascade reductive synthesis of isoindolinones. **(c)** Cascade reductive synthesis of pyrrolidines. **(d)** Synthesis of Xenovenine epimer by double reductive amination. **(e)** Synthesis of pyrrolizidinone PDE 4 inhibitor by cascade reductive recyclization of nitronate. **(f)** Cascade reductive synthesis of tetrahydroquinolines. **(g)** Cascade reductive synthesis of hexahydropyridoquinolines.

The application of polyfunctionalized substrates in the reductive alkylation of nitro compounds provides a versatile access to useful saturated and partially saturated heterocyclic products (characteristic examples are shown in Figure 2). Thus, reductive coupling of o-dinitrobenzene with benzaldehyde over Au/TiO_2_-R catalyst afforded 2-phenylbenzimidazole in 87% yield as reported by Cao and co-authors (Zhang et al., 2016) (Figure 2a). Reductive benzylation of nitroarenes with 2-formylbenzoic acid produced isoindolinones upon catalysis by Au-Pd/Fe_3_O_4_ (Cho et al., 2018) (Figure 2b). This cascade transformation involves the hydrogenolysis of the nitro group, reductive amination reaction with the aldehyde, and subsequent lactamization.

Aliphatic γ-nitrocarbonyl compounds are of particular interest in this context, as their reduction results in the formation of polysubstituted pyrrolidines (Figure 2c), which are a highly important class of saturated heterocycles for drug development (Tasker et al., 1997; Jae et al., 2001; Dziki et al., 2006; Trost and Hisaindee, 2006; García-García et al., 2008; Ballini and Petrini, 2009; Dong et al., 2010, 2019; Xu et al., 2010; Cirujano et al., 2013; Corbett et al., 2014; Sasaki et al., 2014; Emma et al., 2016; Fidalgo et al., 2016). In most of these reactions, standard hydrogenation catalysts like Pd/C, Raney nickel and Adam's catalyst were used. The stereochemical outcome of the intramolecular reductive amination can be controlled by the nature of hydrogenation catalyst (Sukhorukov et al., 2012), albeit the diastereoselectivity is moderate in many cases (Zhu et al., 2010; Lu et al., 2012). The initial γ-nitrocarbonyl compounds are readily accessed by the Michael addition of nitroalkanes to α,β-unsaturated carbonyl compounds (Ballini et al., 2005) or by the addition of aldehydes/ketones to conjugated nitroalkenes (Barrett and Graboski, 1986; Berner et al., 2002) (Figure 2e). Importantly, by using asymmetric organocatalysis, γ-nitrocarbonyl compounds can be prepared in an enantioenriched form, thus allowing access to individual stereoisomers of final pyrrolidines (Sukhorukov et al., 2016; Alonso et al., 2017).

Introduction of a second carbonyl group allows the construction of saturated bicyclic systems containing two fused pyrrolidine units (Ballini and Petrini, 2009; O'Connell et al., 2012). For example, an epimer of alkaloid Xenovenine was synthesized in good yield and stereoselectivity by the catalytic hydrogenation of the γ-nitrodiketone over Pd/C (Vavrecka et al., 1991) (Figure 2d). In this process, two sequential reductive amination reactions lead to the formation of a fully unsymmetrically substituted tertiary amine motif. In another example shown in Figure 2e, the synthesis of a potent PDE4 inhibitor was accomplished by a catalytic cascade recyclization of a cyclic nitronate involving hydrogenolysis of NO_2_ group, intramolecular reductive amination forming a pyrrolidine ring, and subsequent lactamization (Sukhorukov et al., 2012).

Catalytic reduction of δ-nitrocarbonyl compounds results in the formation of a six-membered piperidine ring, which is a common motif in alkaloids and pharmaceuticals. Hydrogenation of 2-nitrocynnamaldehyde over Pt/C or Pd/C, shown in Figure 2f, afforded tetrahydroquinoline in quantitative yield at 100°C and 5 bar H_2_ (Cirujano et al., 2013). Bunce et al. (2001) developed the cascade synthesis of an angular-fused tricyclic amine by hydrogenation of the δ-nitrodicarbonyl compound over Pd-C catalyst (Figure 2g). Intriguingly, in this transformation two new stereogenic centers are formed and the target compound is obtained as a single stereoisomer in 60% yield.

## Discussion and Outlook

Catalytic reductive amination of aldehydes and ketones with nitro compounds is a promising methodology for an efficient and green synthesis of secondary and tertiary amines. To date, numerous catalytic hydrogenation and transfer hydrogenation processes have been designed employing both noble and non-noble metal catalysts. Many of these methods are characterized with high levels of selectivity, broad substrate scope, and functional group tolerance, as well as relatively mild conditions. Reductive amination with nitro compounds was shown to be beneficial for application in the synthesis of various pharmacophore amines and saturated *N*-heterocycles both on laboratory and semi-industrial scales. Nevertheless, there are still many challenges to be addressed in this methodology. Some of these are listed below:

1. Catalysts based on non-noble metals seem to be more efficient compared to noble metals in terms of selectivity and functional group tolerance. However, due to decreased activity and harsh conditions the use of non-noble metals catalysts in reductive alkylation of nitro compounds remains limited.
2. The development of stereoselective methods (in particular, homogeneous catalytic systems) for reductive amination of prochiral ketones with nitro compounds is highly desirable for the application of this methodology in the synthesis of enantiopure active pharmaceutical ingredients.
3. Selective synthesis of fully unsymmetrical tertiary amines by sequential reductive alkylation of nitro compounds is attractive, yet difficult to accomplish in an intramolecular variant.
4. Further design of cascade cyclizations involving reductive amination with nitro compounds would greatly contribute to the synthesis of saturated *N*-heterocycles and polycyclic systems, which are highly relevant to drug development.

Thus, the development of new methods for catalytic reductive amination with nitro compounds can be anticipated in the near future.

## Author Contributions

AS collected the references, summarized the material, and wrote the manuscript.

### Conflict of Interest

The author declares that the research was conducted in the absence of any commercial or financial relationships that could be construed as a potential conflict of interest.
